# Anatomical Injury Clusters in Polytrauma Patients

**DOI:** 10.26502/jsr.10020270

**Published:** 2022-12-15

**Authors:** Tanja Birri, Hans-Christoph Pape, Cyrill Dennler, Hans-Peter Simmen, Jindrich Vomela, Richard Chaloupka, Ladislav Mica

**Affiliations:** 1 Division of Trauma Surgery, University Hospital Zurich, 8091 Zurich, Switzerland; 2 Division of Medical Sciences in Sportsmedicine, Faculty of Sports Studies, Masaryks University, 62500 Brno, Czech Republic; 3 Department of Orthopedic Surgery, Masaryks University, 62500 Brno, Czech Republic

**Keywords:** Polytrauma, AIS, ISS, Truncal Trauma, Retrospective Cohort Study

## Abstract

Polytrauma is a major cause of death in young adults. The trial was to identify clusters of interlinked anatomical regions to improve strategical operational planning in the acute situation. A total of 2219 polytrauma patients with an ISS (Injury Severity Score) ≥ 16 and an age ≥ 16 years was included into this retrospective cohort study. Pearson’s correlation was performed amongst the AIS (Abbreviated Injury Scale) groups. The predictive quality was tested by ROC (Receiver Operating Curve) and their area under the curve. Independency was tested by the binary logistic regression, AIS ≥3 was taken as a significant injury. The analysis was conducted using IBM SPSS^®^ 24.0. The highest predictive value was reached in the combination of thorax, abdomen, pelvis and spine injuries (ROC: abdomen for thorax 0.647, thorax for abdomen 0.621, pelvis for thorax 0.608, pelvis for abdomen 0.651, spine for thorax 0.617). The binary logistic regression revealed the anatomical regions thorax, abdomen pelvis and spine as per-mutative independent predictors for each other when a particular injury exceeded the AIS ≥3. The documented clusters of injuries in truncal trauma are crucial to define priorities in the polytrauma management.

## 1. Introduction

Polytrauma management is still a difficult task. Missed injuries contribute to prolonged hospital stay, higher morbidity and even mortality [1]. For initial use, life support according to ATLS (*Advanced Trauma Life Support*^®^) is the first priority. The adequate treatment of polytrauma patients requires a comprehensive diagnostic work-up [2]. Obviously, missed injuries may cause serious problems [3]. Whole-body-CT scan for primary diagnostics is regarded a gold standard, when readily available. Thus, the application of whole-body-CT scan in hemodynamically unstable severely injured patients seems to be safe, feasible and justified when performed quickly within a well-structured environment and by a well-organized trauma team [4–6]. Hence, the treatment priorities are organized according to injuries found: Treat first, what kills first, afterwards step by step according to ATLS [1]. The surgeons’ decision making should include a comprehensive survey of all organ systems incorporating the patients medical history if needed [7]. The presented study tried to figure out organ injury patterns that may be expected in polytrauma patients suffering a blunt trauma. The knowledge of injury clusters may improve the outcome and lower the complications caused by missed injuries.

## 2. Methods

### 2.1. Patient sample

2189 patients with polytrauma in the years 1996–2017, admitted to the trauma bay of the University Hospital Zurich (Switzerland), were included in this retrospective cohort study. The inclusion criteria were an injury severity score (ISS) ≥16 points, and age ≥16 years. The patients sample was divided into eight groups according to the abbreviated injury scale (AIS) 1–6 of each anatomical region (the AIS of neck and spine were taken together). The accession to this databank was permitted for only the senior author (LM). All patients were subjected to a whole-body-CT scan immediately after admission to the trauma bay if hemodynamically reasonable. All trauma diagnostics depended on the whole-body-CT scan. Patients suffering a cardiac arrest at admission before the whole-body CT scan *were not* included into this study, patients who suffered a cardiac arrest after the whole-body CT scan were included into this study.

### 2.2. Data collection

The study was approved by local institutional review board (IRB) as well as by the ethics committee (Kantonale Ethikkommission *“Retrospektive Analysen in der Chirurgischen Intensivmedizin”* Nr. St.V. 01–2008 and BASEC: 2021–00391). All data were collected retrospectively according to Good Clinical Practice Guidelines and were pooled in the Zurich Trauma Registry Data Bank. All data were fully anonymized and a general consent for further scientific use was obtained, issued by trhe university Hospital of Zurich. The data were analysed for AIS (Abbreviated Injury Scale). Level of Evidence 2b.

### 2.3. Scoring systems

The overall physiological impairment was evaluated by the acute physiology and chronic health evaluation (APACHE II) score at admission [8]. The Injury Severity Score (ISS) and the new injury severity scale (NISS) were used to define the severity of trauma [9,10]. The AIS (update 2015 version) was used to describe injuries in specific anatomical regions. The Trauma Score - Injury Severity Score (TRISS) was used to analyze the probability of death in the patients at admission [11]. Prothrombin time and Hematocrite was measured by the Laboratory of the University Hospital of Zurich.

### 2.4. Statistical analysis

Sets with missing data were excluded. Data are presented as mean ± standard deviation for continuous variables and as percentages for categorical variables. AIS, ISS and NISS were given as median and interquartile range (IQR). Pearson’s correlation was performed according to the different AIS values and tested for significance. A result was considered as significant, if p <.05 (two tailed). The predictive quality of the AIS was reported as the area under the receiver operator characteristic curve (AUC). Independent predictors were determined by binary logistic regression. The goodness of fit for the binary logistic regression was analyzed by the Hosmer-Lemeshow test and considered as good if p > .05. Data were analyzed using IBM SPSS^®^ Statistics for Windows software (version 24.0; IBM Corp., Armonk, NY, USA). Relative density estimation was performed using SigmaPlot 13.0 (Systat Software GmbH, Erkrath, Germay).

## 3. Results

### 3.1. Patient sample

The overall mean age was 44.5 ± 19.6 years and the male to female ratio was 3.0 in a sample of 2189 polytrauma victims (table 1). The overall analysis of the patient data revealed high ISS (29; IQR 25–41) dominated by the AIS head (4.0; IQR 2–6) (table 1). All patients suffered blunt trauma. There are no patients included with gun or stabbing injuries, such patients are lacking in the study’s geographical location. Vehicle accidents, pedestrian traffic accidents and fall from height were the dominating trauma mechanisms.

### 3.2. Correlation, predictive quality and independent predictors: truncal injuries

Pearson’s correlation revealed mainly positive and significant correlation within truncal injuries (thorax, abdomen, pelvis and spine). (table 2) The strongest correlation was found in pelvis and abdomen (0.252) and pelvis correlated to injured extremities (0.259) (table 2). The highest predictive quality was found in abdominal injuries for thoracic injuries (AUC: 0.654), pelvic for abdominal injuries (AUC: 0.630) and pelvic for extremities injuries (AUC: 0.636) (table 3). The predictive independency for each injured truncal region was confirmed by binary logistic regression. Each truncal region was an independent predictor for the other truncal regions except pelvis for spine and spine for pelvis (table 4).

### 3.3. Three dimensional density mapping: Pelvic injuries predict cavitary injuries

**Figure 1:**
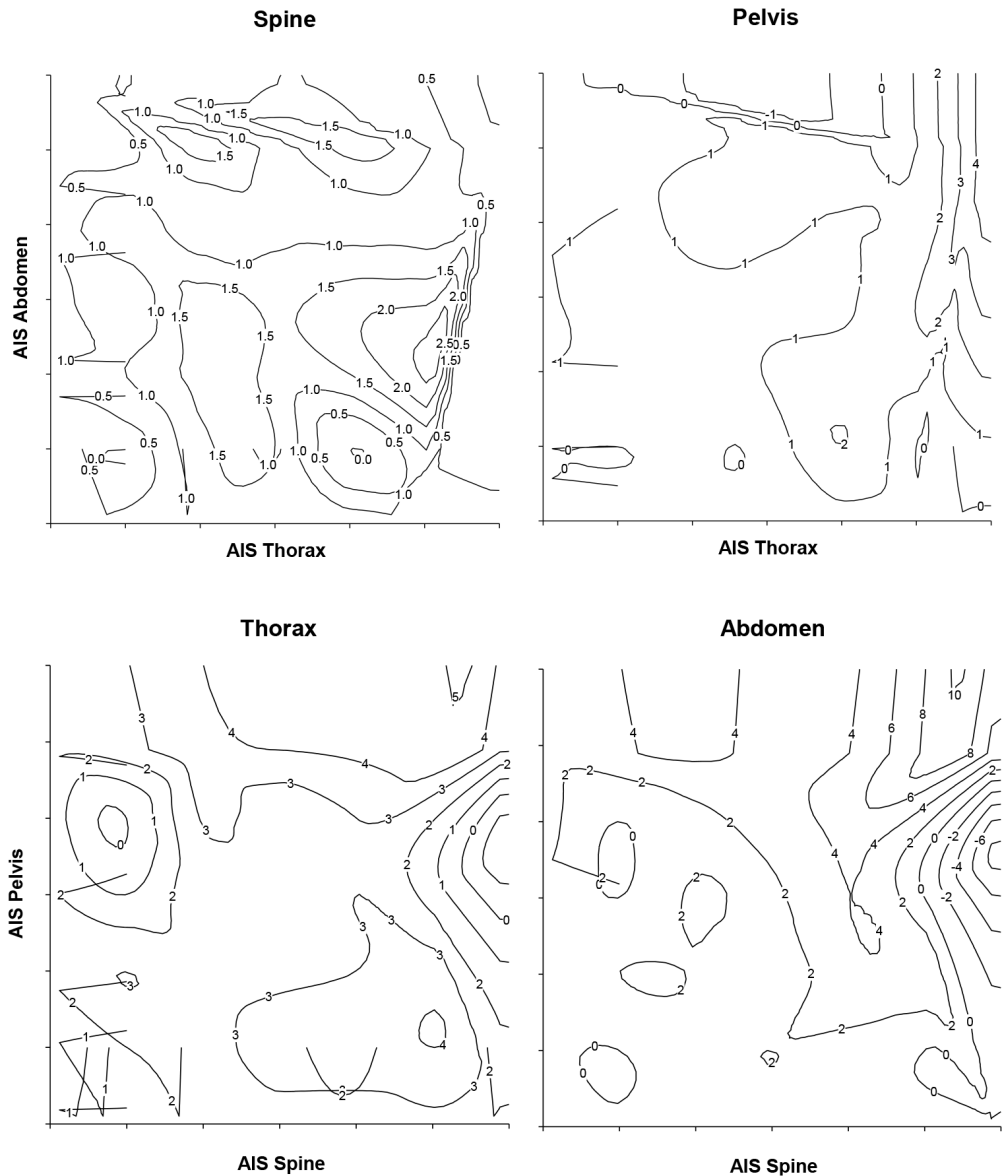
Relative density estimation of the injury-clusters depicted by contour plots. The association between abdominal injuries and spinal injuries is evident (A). Only the highly scored abdominal and thoracic injuries led to a concomitant pelvic injury (B). Spinal injuries are broadly associated with thoracic injuries (C), and pelvic injuries with abdominal injuries. Data are given as artificial units representing relative density of the respective event.

The internal relationship between the injured regions was analyzed by three dimensional density mapping. Severe thoracic injuries in combination with abdominal injuries (AIS < 3) are associated with spinal injuries (figure 1A). However, significant abdominal and thoracic injuries are associated with pelvic injuries (figure 1B). Mostly spinal injuries are associated with thoracic injuries (figure 1C) and severe pelvic injuries (AIS >3) are associated with abdominal injuries especially if in combination with a significant spinal injury (AIS > 3) (figure 1D).

## 4. Discussion

Polytrauma management requires complex interdisciplinary comprehensive medical approach [12–14]. In this retrospective single trauma-center level 1 study in Switzerland the focus was set on the transitions of blunt injuries. All these patients included into this study suffered vehicle accidents and the second etiology was high velocity sports trauma. Missed injuries in a polytrauma patient cause an under treatment or an incorrect treatment strategy and contribute to a higher morbidity and mortality [1]. Certainly, in such situations the whole-body-CT scan is the gold standard [15]. Assumptions about the injury pattern might be drawn from trauma history with a high incertainty. In Pearson’s Correlation, a positive and significant correlation was found between the truncal injuries, soft tissue injuries and injuries of the limbs. High correlations are good, but noteworthy is the predictive quality to look for combined injuries in additional anatomical regions. In this study truncal injuries revealed as independent predictors for each other. Statistical analysis of this study confirms that transitions of injuries in a polytraumatized patients are found in significant truncal injuries with an AIS > 3. Injuries of thorax and abdomen seem to be combined with spinal injuries, in a pelvic injury should be looked for in severe abdominal or thoracic trauma. Pelvic lesions are more indicative for additional abdominal injuries, whereas spinal injuries are often associated with thoracic injuries. In patients suffering from truncal injuries it should be looked for spinal lesions. In a severe abdominal trauma, pelvic and/or spinal injuries have to be taken in account and *vice versa*. Multiple studies have been performed to this topic, however not showing the interdependency of the injury pattern in a polytrauma patient or quantifying the trauma-load [16,17]. However, here starts the inter-observer bias in the evaluation of polytrauma patients. Several studies have been on this topic published, pointing on a very heterogeneous judgement even by the experts in this field [18–20]. The point when a thoracic injury turns into a spinal injury, for example, is not that clear. The points of anatomical injury transition remain undefined. The anatomical transitions should be definitively taken in account by the treating surgeon in the sense of choosing the appropriate surgical approach keeping in mind that the trauma –CT does not depict the complete truth. Exemplary treating a vertical shear pelvic injury (AIS Pelvis 4–5, figure 1A–1D) an early total care concept with screws, plating and intra-peritoneal pelvic tamponade after lower abdominal exploration could be more advisable than retroperitoneal packing and a c-clamp. Anyone knows that missed injuries definitively prolong the hospitalization and lower the overall outcome in polytrauma patients, especially abdominal injuries. The presented results could provide also an orientation for strategical operational planning in these complex patients. In the international point of view, these findings speak clear and uniform language: Whole-body-CT scan is definitively justified in the case of a multiple injured patient [6,15,21–23]. Missed injuries, especially abdominal injuries lead to increased morbidity and mortality in the further treatment course of a polytrauma patient [18]. A clear and evidenced diagnosis in the case of a polytrauma patient definitively improves its outcome. Here, only a small guide was presented with clustering of injuries to guide trauma surgeon’s eye analyzing the whole-body-CT scan and to make strategical planning of acute operations in polytrauma patients.

## 5. Conclusion

Whole-body-CT scan for primary diagnostic work-up is regarded a gold standard to look for isolated as well as combined injuries. Characteristic combinations are: pelvic and intra-abdominal injuries, spinal and thoracic injuries and may guide trauma surgeons’ and radiologists’ attention to the respective anatomical region. The improvement is certainly not the whole-body-CT scan but the higher alertness to combined injuries initially only suspected in the whole-body-CT scan. This might accelerate decision finding in the trauma bay and in the intensive care unit, especially concerning combined abdominal and thoracic injuries. Additionally, a little guide for strategical operational planning in the acute setting might be provided. However, life support according to ATLS (*Advanced Trauma Life Support*^®^) is the first priority.

## 6. Limitations of the study

The main limitation in this study might root in the AIS scoring, hence not automatically/digitally performed, but by different investigators. This inter-observer bias might lead to a background noise in the precision of this analysis [18–20].

## Figures and Tables

**Table 1:** Characteristics of the analyzed patients.

	Median (IQR)
n	2219
Age [a]	44.0 ± 19.6 (Mean ± SD)
Sex [n] (male/female)	1642/577
AIS head	4.0 (1–5)
AIS face	0.0 (0–1)
AIS thorax	3.0 (0–3)
AIS abdomen	0.0 (0–3)
AIS pelvis	0.0 (0–0)
AIS spine	0.0 (0–2)
AIS extremities	2.0 (0–3)
AIS skin	0.0 (0–1)
ISS	29 (25–41)
NISS	41 (34–57)
APACHE II	14.9 ± 8.9 (Mean ± SD)
TRISS	0.75 ± 0.28 (Mean ± SD)
ASCOT	0.29 ± 0.29 (Mean ± SD)
Death within 72h [%]	25

Data are given as Median and Inter Quartile Range, for the age, APACHE II, TRISS and ASCOT as Mean ± SD

**Table 2:** Pearson’s correlation between the different anatomical AIS regions.

Pearson	AIS head	AIS face	AIS thorax	AIS abdomen	AIS pelvis	AIS spine	AIS extremities	AIS skin
*p-value*								
AIS head		**0.143**	**−0.313**	**−0.378**	**−0.218**	**−0.149**	**−0.299**	**−0.088**
		***<.001***	***<.001***	***<.001***	***<.001***	***<.001***	***<.001***	***<.001***
AIS face	**0.143**		0.058	−0.128	−0.024	**−0.06**	0.011	**0.048**
	***<.001***		*0.007*	*<.001*	*0.263*	***0.005***	*0.625*	***0.025***
AIS thorax	−0.313	**0.58**		**0.267**	**0.187**	**0.234**	**0.217**	**0.075**
	*<.001*	***0.007***		***<.001***	***<.001***	***<.001***	***<.001***	***<.001***
AIS abdomen	−0.378	−0.128	**0.267**		**0.253**	**0.062**	**0.132**	**0.069**
	*<.001*	*<.001*	***<.001***		***<.001***	***<.001***	***<.001***	***<.001***
AIS pelvis	−0.218	−0.024	**0.187**	**0.253**		**0.026**	**0.243**	**0.069**
	*<.001*	*0.263*	***<.001***	***<.001***		***<.001***	***<.001***	***<.001***
AIS spine	−0.149	**−0.06**	**0.234**	**0.062**	0.026		**0.049**	0.022
	*<.001*	***0.005***	***<.001***	***0.004***	*0.222*		***0.021***	*0.304*
AIS extremities	−0.299	0.011	**0.217**	**0.132**	**0.243**	**0.049**		−0.135
	*<.001*	*0.625*	***<.001***	***<.001***	***<.001***	***0.002***		*<.001*
AIS skin	−0.088	**0.048**	**0.075**	**0.069**	**0.069**	0.022	−0.135	
	*<.001*	***0.025***	***<.001***	***<.001***	***<.001***	*0.304*	*<.001*	

Highlighted (bold) are the positive and significant correlations.

**Table 3:** Predictive quality tested binary by ROC. Predictive quality of an anatomical region with a significant injury (left row AIS ≥3) for a concomitant significant injury (line, AIS ≥3) in another anatomical region.

AUROC	AIS head	AIS face	AIS thorax	AIS abdomen	AIS pelvis	AIS spine	AIS extremities	AIS skin
AIS head		*0.542*	*0.328*	*0.218*	*0.34*	*0.403*	*0.326*	*0.328*
AIS face	*0.589*		*0.483*	*0.435*	*0.493*	*0.472*	*0.485*	*0.461*
AIS thorax	*0.339*	*0.424*		***0.647***	***0.608***	***0.617***	*0.585*	*0.543*
AIS abdomen	*0.313*	*0.426*	***0.621***		***0.651***	*0.522*	*0.567*	*0.564*
AIS pelvis	*0.407*	*0.479*	*0.577*	***0.605***		*0.494*	***0.603***	*0.553*
AIS spine	*0.436*	*0.462*	***0.601***	*0.533*	*0.522*		*0.52*	*0.5*
AIS extremities	*0.348*	*0.469*	***0.622***	*0.578*	***0.658***	*0.532*		***0.652***
AIS skin	*0.474*	*0.512*	*0.53*	*0.526*	*0.538*	*0.498*	***0.548***	

AUROC > 0.600 is highlighted in bold. To be red as lines from left to right e.g. AIS Thorax for AIS Abdomen.

**Table 4:** Binary logistic regression of the AIS regions with the best predictive quality for concomitant injury.

p-value	AIS thorax	AIS abdomen	AIS pelvis	AIS spine
odds -ratio				
AIS thorax		*<.001*	*<.001*	*<.001*
		*1.388*	*1.285*	*1.287*
AIS abdomen	*<.001*		*<.001*	*0.144*
	*1.178*		*1.345*	*1.044*
AIS pelvis	*<.001*	*<.001*		*0.406*
	*1.366*	*1.46*		*0.963*
AIS spine	*<.001*	*<.001*	*0.009*	
	*1.361*	*1.089*	*1.023*	

Regression was performed from left to right. Hosmer- Lemeshow p < .001. To be red as lines from left to right: e.g. AIS Thorax for AIS Abdomen.
